# Intraobserver and Interobserver Reproducibility of Classifications of Tibial Plateau Fractures and the Surgical Approaches Chosen Comparing 2D CT and 3D Printing: Reliability Study

**DOI:** 10.5435/JAAOSGlobal-D-22-00202

**Published:** 2023-04-11

**Authors:** Jose Osma-Rueda, Fabian Mantilla-Angarita, Clara Lopez-Gualdron

**Affiliations:** From the Faculty of Health Sciences, Universidad Industrial de Santander, Sociedad Colombiana de Cirugía Ortopédica y Traumatología (SCCOT), Bucaramanga, Colombia (Dr. Osma and Dr. Mantilla), and the Industrial Design College, Universidad Industrial de Santander, Bucaramanga, Colombia (Dr. López).

## Abstract

Reliability is the study of internal consistency, reproducibility (intraobserver and interobserver), and agreement. Reproducibility studies that classify tibial plateau fractures have used plain radiography and two-dimensional (2D) CT scans and three-dimensional (3D) printing. The objective of this study was to evaluate the reproducibility of the Luo Classification of tibial plateau factures and the surgical approaches chosen for these fractures based on 2D CT scans and 3D printing.

**Methods:**

A reliability study was performed at the Universidad Industrial de Santander, Colombia, that evaluated the reproducibility of the Luo Classification of tibial plateau fractures and the choice of surgical approaches based on 20 CT scans and 3D printing, with five evaluators.

**Results:**

For the trauma surgeon, reproducibility was better when evaluating the classification using 3D printing, with a kappa of 0.81 (95% confidence interval [CI], 0.75-0.93; *P* < 0.01) than when using CT scans, with a kappa of 0.76 (95% CI, 0.62-0.82; *P* < 0.01). When comparing the surgical decisions made by the fourth-year resident with those of the trauma surgeon, a fair reproducibility was obtained using CT, with a kappa of 0.34 (95% CI, 0.21-0.46; *P* < 0.01), which improved to substantial when using 3D printing, with a kappa of 0.63 (95% CI, 0.53-0.73; *P* < 0.01).

**Discussion:**

This study found that 3D printing provided more information than CT and decreased measurement errors, thereby improving reproducibility, as shown by the higher kappa values that were obtained.

**Conclusion:**

The use of 3D printing and its usefulness are helpful to decision making when providing emergency trauma services to patients with intraarticular fractures such as those of the tibial plateau.

Tibial plateau fractures of the knee represent approximately 1% of all fractures^1^ and 8% of all fractures in adults.^2^ Fractures on the surface of the tibial plateau present the risk of biomechanical instability of the knee articulation.^3,4^ Therefore, a diagnosis that helps to make decisions about the effective treatment of these fractures is crucial.^5^ Nevertheless, when complex intraarticular fracture patterns occur in the area of the tibial plateau, it is not always easy to identify the fracture lines.^6^ This can result in performing diagnostics and/or surgical approaches that are not indicated. It is important that orthopedic and trauma surgeons have better information for making decisions, which can come from three-dimensional (3D) printing models.

An entirely reliable classification for this type of fracture does not currently exist, which is why we suggest that the use of 3D printing can decrease measurement errors in reliability.

Classifications of fractures should be validated, including tibial plateau fractures. This includes content and construct validation.^7-9^

Reliability is studied based on three factors: internal consistency, reproducibility, and agreement. Intraobserver and interobserver reliability of the classification of tibial plateau fractures (reproducibility) has used plain radiography, two-dimensional (2D) CT scans, and 3D reconstruction, with increasingly better reliability values being obtained since the technological evolution of the imaging has improved the information that the evaluator and the clinician depend on to make decisions. This has resulted in better clinical outcomes for patients and improved reliability.^5,7,9^

With the technological advances that have been made in medicine, some studies have combined imaging techniques such as CT and new technologies such as 3D printing for the reconstruction of anatomical models.^10-12^ Just as with the evolution of information from diagnostic images, 3D printing provides better and more real information, which helps to reach more precise diagnoses based on precise anatomical models.^12^ As shown by prior evidence, 3D printing can be used to visualize the characteristics of tibial plateau fractures on axial, sagittal, and coronal planes.

The objective of this study was to evaluate intraobserver and interobserver reliability of the Luo Classification of tibial plateau fractures^13^ and the choice of surgical approaches for these fractures by comparing 2D CT scans with 3D printing.

## Methods

**Figure 1:**
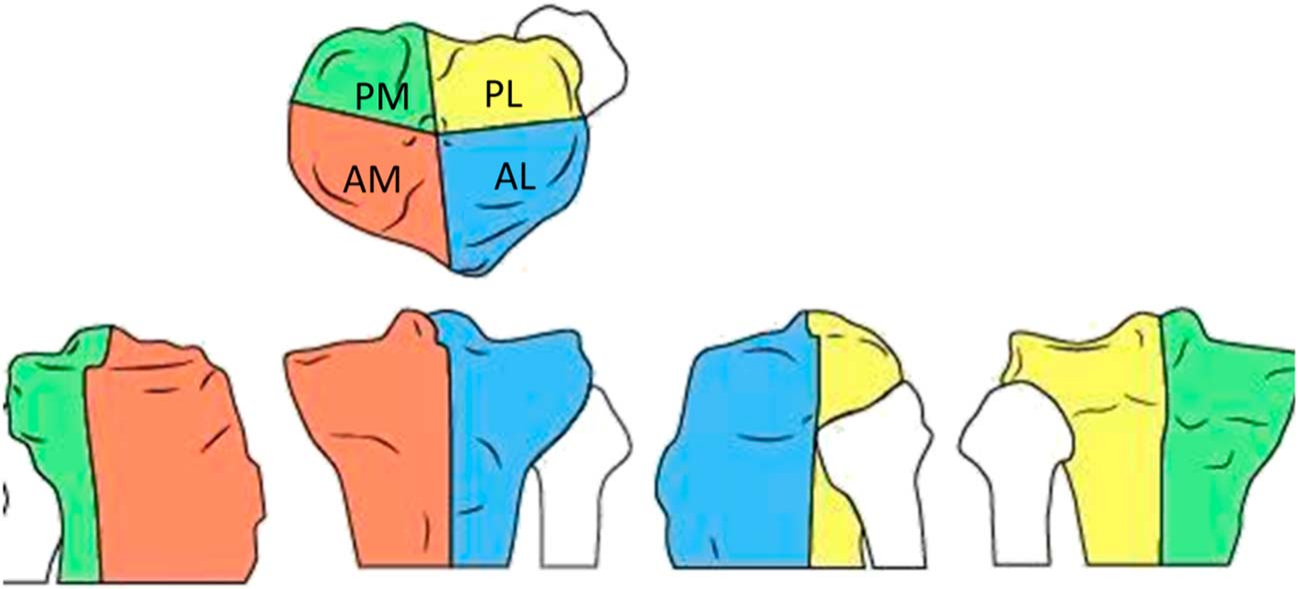
Illustration showing the classification by Luo et al. of the plateu fractures.^13^ AL = Anterolateral, AM = Anteromedial, PL = Posterolateral, PM = Posteromedial.

With approval from the Ethics and Research Committee of the Universidad industrial de Santander and the Hospital Universitario de Santander in Bucaramanga, Colombia, an interobserver and intraobserver reliability study was performed between the years 2017 and 2019 to assess reproducibility when evaluating the Luo Classification^13^ of tibial plateau fractures and making decisions about the surgical approaches for each of the cases evaluated (Figure 1). For this purpose, the study used CT scans of different types of tibial plateau fractures from 20 patients not randomized. The inclusion criteria were patients older than 18 years of age who had an unintentional injury, as injuries caused by transit or injuries caused by dairy activities. Patients with pathological fractures, injuries caused by gun weapons, blunt trauma, and open fractures were excluded.

**Figure 2:**
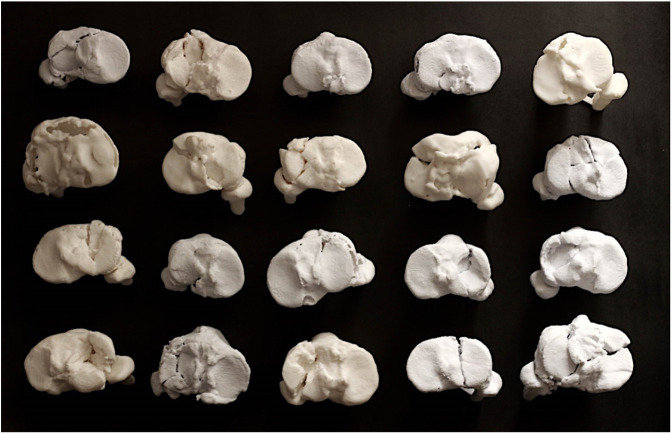
Illustration showing the 3D-printing biomodels used in the study.

The images of the fractures were obtained with CT image segmentation. The CT scans were generated with an Aquilion 64-slice helical system with 0.5 mm slices of the tibial plateau and were stored with digital imaging and communications in medicine. The 3D printing models of the 20 cases shown in Figure 2 were obtained with free reverse engineering software known as InVesalius,^14^ which specializes in generating virtual 3D imaging. These were prepared with computer-aided design software, after which 3D models were printed at actual scale in polylactic acid using fused deposition modeling, with a resolution of 0.1 mm. The Interfaz group conducted the process for generating the biomodels at the 3D technology laboratory at the Universidad Industrial de Santander.^15,16^

Five evaluators were included in this study: three orthopaedic surgeons and traumatologists, one specialized in trauma surgery, one knee surgeon, and one general orthopaedic surgeon; and two students of a third-year and a fourth-year resident of orthopedy and traumatology who had previously reviewed the Luo Classification^13^ and the surgical approaches, namely anterolateral, anteromedial, midline, posteromedial, posterolateral, posterior inverted L, and minimally invasive. A pilot test was performed with three cases to become familiar with the data collection instrument, the classification, and the surgical approaches used for tibial plateau fractures.

The 5 evaluators assessed the 20 cases blindly, independently, at different times, and randomized. The classifications and choice of surgical approaches were evaluated for each case according to the type of fracture, based on the Luo Classification of the four tibial plateau columns (Figure 1).^13^ Each evaluator assessed the 2D CT and 3D printing of each case under the same conditions, every 2 weeks, but the durations of the evaluation time was not standardized.

### Stages and Times for Evaluating the 2D CT Scans and 3D Printing Models

Next, the activities that were performed will be described and the time during the study when they were conducted, with a difference of 2 weeks between the first and second assessment for CT and 3D, evaluating classification and surgical approach.

#### First

The CT images that were collected and printed of the tibial plateaus were reviewed to evaluate the classifications.

#### Second

Two weeks after the first evaluation, the 3D printing models were reviewed to evaluate the classifications.

#### Third

Four weeks after the first evaluation, another assessment was performed of the CT scan images to choose the surgical approaches of the tibial plateau fractures.

#### Fourth

Six weeks after the first evaluation, a second assessment was performed of the classifications and choice of surgical approaches based on each of the 3D printing models of the tibial plateau fractures.

### Information Processing and Analysis Statistics

The information obtained with the data collection instruments was entered in duplicate in an Excel spreadsheet, and the data were processed using STATA version 16 software. A descriptive analysis was performed of the sociodemographic characteristics of the patients included in the study and the frequency distributions. The continuous variables according to the number of patients included (n = 20) and the distribution are presented in medians and interquartile ranges.

Reproducibility was evaluated with the kappa statistic and their 95% confidence interval (CI),^17,18^ which were interpreted based on the proposal by Landis and Koch^19^: 0.01 to 0.20 slight, 0.21 to 0.40 fair, 0.41 to 0.60 moderate, 0.61 to 0.80 substantial and 0.81 to 1.00 almost perfect.

## Results

A total of 200 evaluations were performed of intraobserver reliability for each evaluation method: 2D CT and 3D printing. Another 200 evaluations were conducted of interobserver reliability for the surgical approaches that were selected for the different types of tibial plateau fractures based on the CT scans and 3D printing.

### Sociodemographic Characteristics

In the main overall characteristics of the 20 patients in the study, the median age was 34 years (18-78) and most were men (18 of 20). The left side was most often affected, with 11 patients, and the most frequent mechanism of injury was trauma caused by traffic accidents (17 patients), with motorcycle crashes being the most common (13 patients).

### Intraobserver Reproducibility of the Classification With CT Scans

In the intraobserver reproducibility of the Luo Classification of the tibial plateau fractures when using CT scans, based on Landis and Koch,^19^ the assessment by the trauma surgeon was substantial, with a kappa of 0.76 (95% CI, 0.62-0.82; *P* < 0.01), followed by the knee surgeon and the fourth-year resident who obtained a kappa of 0.69 (95% CI, 0.60-0.80; *P* < 0.01) (Table 2). In the surgical approaches chosen based on reviewing the CT scans, the intraobserver reproducibility for the trauma surgeon was almost perfect, with a kappa of 0.69 (95% CI, 0.60-0.80; *P* < 0.01) and 85% agreement, followed by the knee surgeon with a substantial kappa of 0.67 (95% CI, 0.57-0.76; *P* < 0.01) and 75% agreement (Table 1).

**Table 1:** Intraobserver Reliability With 2D CT: 3D Printing for the Luo Classification and Surgical Approach

Evaluator	CT/Classification			3D/Classification			CT/Approach			3D/Approach		
	Kappa	95% CI	%	Kappa	95% CI	%	Kappa	95% CI	%	Kappa	95% CI	%
Trauma surgeon	0.76^a^	0.62-0.82	80	0.81^a^	0.75-0.93	85	0.81^a^	0.79-0.93	85	0.93^a^	0.91-1.00	95
Knee surgeon	0.69^a^	0.60-0.80	75	0.53^a^	0.47-0.64	60	0.67^a^	0.59-0.76	75	0.58^a^	0.41-0.70	65
Orthopaedic surgeon	0.29^a^	0.16-0.37	45	0.49^a^	0.41-0.53.	60	0.59^a^	0.37-0.63	75	0.76^a^	0.38-0.92.	85
Third-year resident	0.55^a^	0.43-0.71	60	0.50^a^	0.36-0.60.	60	0.50^a^	0.25-0.82	55	0.51^a^	0.45-0.55	65
Fourth-year resident	0.65^a^	0.50-0.73	70	0.27^a^	0.07-0.38	35	0.54^a^	0.38-0.62	65	0.80^a^	0.55-0.92	85

2D = two-dimensional, 3D = three-dimensional printing, CI = confidence interval

a *P* < 0.01.

### Intraobserver Reproducibility of the Classification With 3D Printing

In respect of the classification of tibial plateau fractures, intraobserver reproducibility with 3D printing was almost perfect for the trauma surgeon, with a kappa of 0.81 (95% CI, 0.75-0.93; *P* < 0.01) (Table 1).

### Intraobserver Reproducibility of Classifications and Surgical Approaches Using 3D Printing

Furthermore, reproducibility for the group of evaluators was better with 3D printing than with 2D CT scans. Reproducibility of the choice of surgical approach was also better with 3D printing for the entire group of evaluators, and the trauma surgeon continued to be better, having obtained an almost perfect result with a kappa of 0.93 (95% CI, 0.92-1.00; *P* < 0.01) (Table 1).

### Interobserver Reproducibility of Classifications and Surgical Approaches Using 3D Printing

When using 3D printing, interobserver reliability was slight for the classification, with a kappa of 0.18 (95% CI, 0.12-0.19), and moderate for the surgical approach, with a kappa of 0.47 (95% CI, 0.36-0.56). Both were statistically significant (*P* < 0.01).

Finally, when analyzing the interobserver reproducibility of the surgical approach by comparing the more experienced trauma surgeon with the other evaluators, reproducibility was better with the 3D printing for both the Luo Classification^13^ and the surgical approach (Table 2).

**Table 2:** Interobserver Reliability With 2D CT: 3D Printing for the Luo Classification and Surgical Approach Compared With the Trauma Surgeon

Evaluator	CT/Classification			3D/Classification			CT/Approach			3D/Approach		
	Kappa	95% CI	%	Kappa	95% CI	%	Kappa	95% CI	%	Kappa	95% CI	%
Knee surgeon	0.24^a^	0.14-0.36	35	0.30	0.17-0.41	40	0.32	0.21-0.38	45	0.56	0.56-0.67	65
Orthopaedic surgeon	0.25^a^	0.14-0.43	35	0.44	0.29-0.66.	55	0.24	0.16-0.35	40	0.36	0.27-0.49	50
Third-year resident	0.22^a^	0.19-0.43	30	0.10	0.03-0.17	20	0.20	0.10-0.31	25	0.27	0.25-0.33	45
Fourth-year resident	0.20^a^	0.10-0.31	30	0.16	0.09-0.23	25	0.34	0.21-0.46	45	0.63	0.53-0.73	70

2D = two-dimensional, 3D = three-dimensional printing, CI = confidence interval

a *P* < 0.01.

## Discussion

This reliability study compared the interobserver and intraevaluator reproducibility of the Luo Classifications of tibial plateau fractures and the corresponding choice of surgical approaches based on 3D printing versus 2D CT scans. When comparing 3D printing and 2D CT scans, the intraobserver reproducibility of the classification and surgical approach was always better with 3D printing for each of the five evaluators, with the surgical approach being even better and statistically significant. In addition, the most experienced evaluator obtained an almost perfect kappa value^19^ for the surgical approach when using 3D printing models. When comparing 2D CT scans and 3D printing for the fourth-year resident, intraobserver reliability of the surgical approach improved from a kappa of 0.54 (95% CI, 0.38-0.62) with the CT scans to a kappa of 0.80 (95% CI, 0.55-0.92) with 3D printing (Table 1).

Regarding interobserver reproducibility, the kappa value was found to be affected by the prevalence of the event and by bias because of experience (paradox bias),^9^ which in this study related to the trauma surgeon who had the most experience and whose evaluations were more reliable than those of the two residents in training. The lack of experience by the residents resulted in slight interobserver reliability values (Table 2).^18^ Under the scenario described, with five evaluators who had different competencies, this could be considered a weakness of the study; however, it is a reality in the daily practice at our services, especially university services.^8,9,18^ Another factor that affects the kappa values is the number of categories to be evaluated, as in the study herein with the classification of fractures and surgical approaches to be selected. In addition, the 20 patients who were studied had different types of tibial plateau fractures (Figure 1). According to the COnsensus-based Standards for the selection of health Measurement INstruments (COSMIN checklist) for reliability studies, the evaluation of 20 patients based on 2D CT scans and 3D printing could be considered to be a small sample and a weakness of the study because a minimum of 50 participants is recommended for reliability studies.^20,21^ Furthermore, because the duration of the evaluation time every evaluator took was not standardized, it would be considered another weakness of this study.

The type of instrument used affects reliability, whether using kappa or the intraclass correlation coefficient. In this study, the use of images such as 2D CT and 3D printing resulted in better reproducibility values than radiography.^5,9,22^ The use of 3D printing models by this reliability study is a strength because it provides better information and aids in decision-making by decreasing interpretation errors.

Reliability studies of the classification of tibial plateau fractures have used instruments such as plain radiography.^13,23,24^ In addition, Schatzker AO/OTA^25^ obtained intraobserver kappa values that were between moderate (0.41-0.60) and substantial (0.61-0.80).^19^ Studies that use 2D or 3D printing improve intraobserver and interobserver reproducibility,^25-27^ with kappa values between slight (0.00-0.20) when evaluating 2D CT scans and moderate (0.41 a 0.60) when evaluating 3D printing models.^19^ Furthermore, the kappa value for intraobserver and interobserver reproducibility has been reported to be better with 3D printing.^7^

Normally, the kappa value for interobserver reliability^17^ is not high for the different classifications of fractures, ranging from 0.3 (fair) to 0.7 (substantial).^9,27^ The interpretation of reliability depends on the context of the evaluator and the instrument used for its evaluation in the clinical practice. In addition, high reliability does not indicate high precision.^8,9^

Innovations in diagnostic imaging have helped with clinical and surgical decisions, which has benefited the treatment of tibial plateau fractures and decreased measurement error in reliability studies.

The 3D printing provides more information, facilitating planning and decision-making for tibial plateau fractures, which are more complex because of the increased energy that they cause. Furthermore, reliability as represented by intraobserver and interobserver reproducibility results in higher and more reproducible kappa using 3D printing coefficient values.

## Conclusion

3D imaging helps to interpret and analyze tibial plateau fractures on the axial, sagittal, and coronal planes by decreasing measurement error.^22^ It also improves intraobserver and interobserver reliability in the classification of these fractures and even more improvement is gained in the surgical approach that is selected. The use of 3D printing and its usefulness are helpful to decision-making when providing emergency trauma services to patients with intraarticular fractures such as those of the tibial plateau.
